# Nicotine Prevents Oxidative Stress-Induced Hippocampal Neuronal Injury Through α7-nAChR/Erk1/2 Signaling Pathway

**DOI:** 10.3389/fnmol.2020.557647

**Published:** 2020-11-12

**Authors:** Yun Dong, Wenchuan Bi, Kai Zheng, Enni Zhu, Shaoxiang Wang, Yiping Xiong, Junlei Chang, Jianbing Jiang, Bingfeng Liu, Zhonghua Lu, Yongxian Cheng

**Affiliations:** ^1^School of Pharmaceutical Sciences, Health Science Center, Shenzhen University, Shenzhen, China; ^2^Shenzhen Institutes of Advanced Technology, Chinese Academy of Sciences, Shenzhen, China

**Keywords:** oxidative stress, nicotine, neuroprotection, ERK1/2, α7-nAChRs

## Abstract

Oxidative stress-induced neuronal damage has been implicated to play a dominant role in neurodegenerative disorders, such as Alzheimer’s disease (AD). Nicotine, a principal additive compound for tobacco users, is thought as a candidate to attenuate amyloid-β-mediated neurotoxicity and NMDA-induced excitotoxicity. Previous studies demonstrated that nicotine exerted this neuroprotective action on oxidative stress. However, the mechanisms underlying how nicotine contributes on oxidative injury in immortalized hippocampal HT-22 cells remain largely unknown. Therefore, in this study we investigated that the potential effects of nicotine on hydrogen peroxide (H_2_O_2_)-induced oxidative injury and underlying mechanisms in HT-22 cells. We found that pretreatment with nicotine at low concentrations markedly recovered the cell cycle that was arrested at the G2/M phase in the presence of H_2_O_2_ through reduced intracellular ROS generation. Moreover, nicotine attenuated H_2_O_2_-induced mitochondrial dysfunctions. Mechanistically, the application of nicotine significantly upregulated the levels of phosphorylated Erk1/2. The neuroprotective effects of nicotine, in turn, were abolished by PD0325901, a selective Erk1/2 inhibitor. Further obtained investigation showed that nicotine exerted its neuroprotective effects *via* specifically activating α7 nicotinic acetylcholine receptors (α7-nAChRs). A selective inhibitor of α7-nAChRs, methyllycaconitine citrate (MLA), not only completely prevented nicotine-mediated antioxidation but also abolished expression of p-Erk1/2. Taken together, our findings suggest that nicotine suppresses H_2_O_2_-induced HT-22 cell injury through activating the α7-nAChR/Erk1/2 signaling pathway, which indicates that nicotine may be a novel strategy for the treatment of neurodegenerative disorders.

## Introduction

Oxidative stress caused by the accumulation of excessive reactive oxygen species (ROS) damages proteins, DNA, and membranes, which thereby disrupts neuronal cell functions and triggers neuronal cell death and eventually leads to neurodegenerative diseases (Cao and Kaufman, 2014; Tasdogan et al., 2016; Debattisti et al., 2017; Valverde et al., 2018). It is well known that the mammalian brain has a high concentration of oxygen but low levels of antioxidant enzymes (Khan and Black, 2003; Olmez and Ozyurt, 2012), suggesting that neurons are particularly vulnerable to ROS-induced oxidative stress. Moreover, oxidative stress is postulated to be a critical factor associated with pathophysiological progression of Alzheimer’s disease (AD; Smith et al., 1996), which, at least in part, contributes to destruction of neurons by amyloid-β (Aβ; Harris et al., 1995).

Hydrogen peroxide (H_2_O_2_), an inducer of highly reactive ROS, is responsible for the majority of oxidative neuronal damage (Behl et al., 1994; Riley, 1994; Desagher et al., 1997). H_2_O_2_ has been widely used as a neurotoxic paradigm to mimic *in vitro* oxidative stress in many different cell types. For instance, H_2_O_2_ caused intracellular ROS generation and repressed mitochondrial membrane potential, which then underwent apoptosis in PC12 cells (Gao J. et al., 2018) and in SK-N-MC cells (Lee and Kim, 2019). Similarly, mitochondrial dysfunctions induced by H_2_O_2_ occurred in HT-22 cells as well (Dai et al., 2014). Evidence further demonstrated that H_2_O_2_-induced mitochondrial membrane depolarization, swelling, and fragmentation could be due to the motility of mitochondria accompanied with mitochondrial elongation (Debattisti et al., 2017). Moreover, evidence showed that H_2_O_2_ could induce autophagic death in dopaminergic SY5Y cells through ROS-dependent endoplasmic reticulum stress and AMPK activation (Gao Z. et al., 2018). Therefore, it is of importance to identify a mechanism that exerts neuroprotective effects against oxidative injury.

Nicotine has been recognized as the principal additive compound of tobacco that causes devastating health problems and even premature death for tobacco users (Hoffmann et al., 1990; Benowitz, 2009; Hatsukami et al., 2008). Nicotine abuse induces oxidative stress, apoptosis, and inflammation in brain cells (Oliveira-da-Silva et al., 2009; Benowitz, 2010; Cardinale et al., 2012; Motaghinejad et al., 2016) and also exacerbates behavioral impairments in mice (Shim et al., 2008). Chronic nicotine administration exacerbates tau pathology in a mouse model of AD (Oddo et al., 2005). Interestingly, frequency of dietary nicotine however has been reported to be inversely associated with Parkinson’s disease (PD) risk (Nielsen et al., 2013). These studies suggest that nicotine might exert opposite roles with respect to neurodegeneration and neuroprotection. Indeed, an experimental study showed that nicotine prevents dopaminergic neuron loss in a rodent PD model (Liu Y. et al., 2017). Evidence has also accumulated that nicotine has been linked with decreased risk for AD (Oddo et al., 2005; Echeverria and Zeitlin, 2012; Moreno-Gonzalez et al., 2013; Lombardo and Maskos, 2015). Nicotine could attenuate Aβ peptide-induced neurotoxicity in hippocampal neurons of rats (Liu and Zhao, 2004). Moreover, an *in vitro* study showed that nicotine is neuroprotective against NMDA-induced excitotoxicity (Dajas-Bailador et al., 2000). The actual results indicate the opposite effects of nicotine in the CNS, neuroprotective effects, and neurotoxic effects. Importantly, nicotine has been reported to encourage oxidative impairments in rat’s brain (Barr et al., 2007; Benowitz, 2010; Saad et al., 2020); nevertheless, increasing studies *in vitro* and *in vivo* showed the functions of nicotine on oxidative stress (Guan et al., 2003; Liu and Zhao, 2004; Hritcu et al., 2017). For instance, nicotine could neuroprotect against oxidative stress in primary cultures (Liu et al., 2015), in PC12 cells (Slotkin et al., 2015). Moreover, antioxidative functions of nicotine have been indicated in SY5Y cells (Parada et al., 2010). However, the contribution of nicotine on oxidative injury and its underlying mechanisms in mouse hippocampal HT-22 cell remain largely unknown.

In the present study, we investigated whether nicotine could mitigate H_2_O_2_-induced oxidative damage in HT-22 cells and explored the potential molecular mechanisms. Thereby, a thorough understanding of the potential functions of nicotine on oxidative stress will be revealed, and this could promote the development of effective agents in the treatment of these conditions.

## Materials and Methods

### Reagents and Antibodies

The FITC-labeled Annexin V Apoptosis Detection Kit was obtained from BD Biosciences (Canada). The ROS assay kit (DCFH-DA) was purchased from Meilun (China). The cell culture medium was obtained from HyClone (Utah, USA), and cell-cultured grade fetal bovine serum (FBS), penicillin/streptomycin, and trypsin were purchased from Gibco (Thornton, Australia). The antibodies of p-Erk1/2, Erk1/2, p-Akt, Akt, cleaved-caspase 3, caspase 3, cleaved-caspase 9, caspase 9, β-actin, and horseradish peroxidase (HRP)-conjugated goat anti-rabbit antibody were obtained from Cell Signaling Technology (Danvers, MA, USA). Anti-nicotinic acetylcholine receptor α7 antibody was purchased from Abcam (ab216485, Abcam). The drugs were obtained from the following sources: nicotine, methyllycaconitine citrate (MLA), dihydro-β-erythroidine hydrobromide (DHβE), and PD0325901 from MedChemExpress (MCE, USA) and H_2_O_2_ and *N*-acetylcysteine (NAC) from Sigma–Aldrich (St. Louis, MO, USA).

### Cell Culture

HT-22 cells (a mouse hippocampal cell line) purchased from iCell company in Shanghai, China, were cultured in Dulbecco’s modified Eagle’s medium (DMEM) with 10% FBS, 100 U/ml penicillin, and 100 U/ml streptomycin. The cells were maintained in a humidified 5% CO_2_ atmosphere at 37°C. The cells were plated at a density of 1 × 10^4^/well in 96-well plates and 2 × 10^5^/well in six-well plates, respectively. After 24 h, cells were applied for various treatments and subjected to measurements.

### Cell Viability

Cell cytotoxicity was quantified by a Cell Counting Kit (CCK-8, Dojindo Laboratory, Kumamoto, Japan), following the manufacturer’s instruction. HT-22 cells were plated in 96-well plates with the application of various drugs. CCK-8 solution (10 μl/well) was added to each well and incubated for an additional 1 h at 37°C in 5% CO_2_. Then, the spectrophotometric absorbance at 450 nm was determined by using a microplate reader (BioTek).

### Cell-Cycle Analysis

For cell-cycle analysis, HT-22 cells were seeded in six-well plates with the application of different drugs, then the cells were washed twice with cold PBS. The cells were resuspended in 300 μl PI/RNase Staining Buffer (BD Biosciences) and incubated for 15 min at room temperature in the dark. The cells were then analyzed by a FACSCalibur flow cytometer at 480 nm. Data were analyzed with CELLQuest software.

### Measurement of Cell Proliferation

HT-22 cell proliferation was determined by EdU incorporation assay *via* EdU cell proliferation Kit with Alexa Fluor 594 following the manufacturer’s instructions (Beyotime, China). HT-22 cells were seeded in six-well plates and were allowed to be treated with various drugs for 24 h. Cells were then incubated with 10 μM EdU solution in DMEM medium for 4 h. The cells were washed with washing buffer (PBS containing 3% BSA), followed by fixation of 4% polyformaldehyde for 15 min and then permeabilization with PBS containing 0.3% Triton X-100 for 20 min. After three times washing, cells were incubated with azide-conjugated Alexa Fluor 594 for 30 min in click addictive reactive buffer with 4 mM CuSO_4_. Cells were then washed three times with washing buffer. DAPI (1:1,000, Beyotime, China) was incubated with cells in PBS solution for 10 min at room temperature. The cells in six different areas of each well were photographed under a fluorescent microscope and analyzed with ImageJ software. The percentage of proliferated cells was calculated as EdU-positive cell number/total cell number × 100%.

### Measurement of Mitochondrial Membrane Potential

Mitochondrial membrane potential has been used as an important parameter of mitochondrial function (Liu et al., 2018). To assess the level of mitochondrial membrane potential, a commercial cyanine dye JC-1 assay kit (5,5′,6,6′-tetrachloro-1,1′,3,3′-tetraethyl-imidacarbocyanine iodide, Beyotime, China) was used according to the manufacturer’s instructions. Previous studies have described that JC-1 staining for mitochondria, either as red fluorescent J-aggregates or as green fluorescent J-monomers, was used for monitoring the mitochondrial membrane potential (Smiley et al., 1991). J-aggregates at higher mitochondrial concentrations reflected higher mitochondrial potential, and J-monomers at lower mitochondrial concentrations indicated lost membrane potential. Accordingly, the J-aggregate/J-monomer (red/green) fluorescence intensity ratio monitored mitochondrial membrane potential fluctuations. In brief, the cells were collected and washed in PBS and then incubated with 1 ml JC-1 (5 μg/ml) staining solution for 20 min at 37°C in dark. Cell treated with 10 μM carbonyl cyanide m-chlorophenylhydrazone (CCCP) was used as positive control. CCCP is a protonophore which can cause dissipation of mitochondrial membrane potential. Subsequently, the cells were measured using flow cytometry. The red fluorescence was measured at the excitation wavelength of 530 nm and the emission wavelength of 590 nm. The green fluorescence was detected at the excitation wavelength of 485 nm and the emission wavelength of 530 nm. The changes in mitochondrial membrane potential were calculated as the JC-1-stained red/green fluorescence intensity ratio, which were analyzed using FlowJo v7.6 software 41.

### Analysis of Apoptosis

HT-22 cells seeded in six-well plates were allowed to be treated with various drugs for 24 h. The apoptosis assay was conducted using Annexin V-FITC/PI apoptosis detection kit (BD Biosciences, Mississauga, ON, Canada) following the manufacturers’ instruction. The cells were collected and washed with cold Ca^2+^-free PBS. Then, the cells were resuspended in 500 μl 1× binding buffer, containing Annexin V/FITC and 5 μl PI for 15 min at room temperature in the dark. Cell apoptosis was analyzed by using a flow cytometer. Data were analyzed using FACSAria equipped with the CellQuest Software.

### Measurement of Intracellular ROS

The amount of intracellular ROS was measured by probe 2′,7′-dichlorodihydrofluorescein diacetate (DCFH-DA, ROS assay Kit, Meilun, China) in HT-22 cells. The treated cells were washed twice with PBS and then incubated with 10 μM DCFH-DA at 37°C for 30 min. After washing with fresh DMEM, cells were collected by trypsin. Cells were analyzed using a flow cytometer (cytoFLEX, Beckman Coulter), and the level of ROS was measured as the mean fluorescence intensity. The images of the cells were captured in six different areas of each well under a fluorescent microscope (AxioVert Al, Zeiss, Germany) and measured by using ImageJ (NH) software. The number of ROS-positive cells was calculated as a percentage: positive cell number/total cell number × 100%.

### Western Blot Analysis

HT-22 cells were seeded in six-well plates and were allowed to be treated with various drugs for 24 h. The cells were rinsed twice with cold PBS and lysed by homogenization of RIPA buffer (Beyotime Institute of Biotechnology, Shanghai, China), and a phosphatase inhibitor and a protease inhibitor cocktail tablet (1:50, Roche, Germany) were added for 30 min on ice. Then, the collected cell lysates were vortexed and the insoluble cell debris were removed using centrifugation at 12,000× *g* for 10 min at 4°C. The total protein concentrations were measured using a Pierce BCA Protein Assay Kit (Thermo Fisher Scientific, Waltham, MA, USA), and then all lysates were diluted to the same concentration. The cell lysates were boiled in a gel-loading buffer at 95°C for 10 min. The protein extracts of 10 μg were separated in 10% acrylamide gel by electrophoresis and then transferred to polyvinylidene fluoride Immun-Blot PVDF Membranes (Bio-Rad, Hercules, CA, USA). Membranes were blocked with 5% skim milk in a Tris-buffered saline containing 0.05% (v/v) Tween-20 (TBS-T) for 1 h at room temperature before an overnight incubation at 4°C with various primary antibodies. The antibodies were in different dilution as follows: p-Erk1/2, Erk1/2, p-Akt, Akt, cleaved caspase-3, caspase-3, cleaved caspase-9, and caspase-9 in 1:1,000 dilution; nAChR α7 in 1:300 dilution; and β-actin in 1:5,000 dilution. Blots were rinsed with TBS-T and incubated for 1 h at room temperature with an HRP-conjugated secondary antibody at a 1:5,000 dilution. Reactive bands were visualized by the Quantity One automatic imaging analysis system (Bio-Rad, Hercules, CA, USA) using enhanced chemiluminescence ECL (Millipore, Kankakee, IL, USA). The intensities of the immune-reactive bands were calculated by ImageJ software.

### Statistical Analysis

Statistical comparisons were performed using one-way analysis of variance (ANOVA) followed by a *post hoc* test. Data were expressed as the mean ± standard error of the mean (SEM). Statistical significance was taken at *p* < 0.05.

## Results

### H_2_O_2_ Inhibits HT-22 Cell Growth

**Figure 1:**
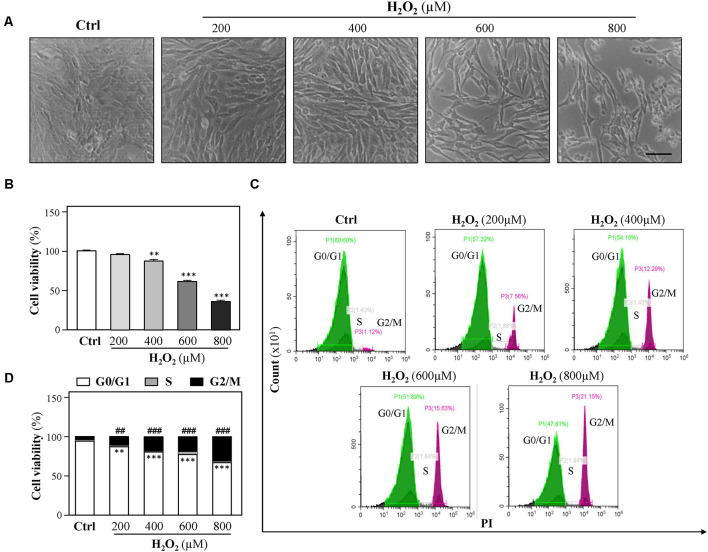
H_2_O_2_ inhibits HT-22 cell proliferation. Cultured HT-22 cells were incubated with H_2_O_2_ at different concentrations (200–800 μM) for 24 h; the control group (Ctrl) consists of the untreated cells. **(A)** Representative images of the cell morphology. Bar = 100 μm. **(B)** Cell viability test was performed using CCK-8 kit. **(C)** The cell cycle was detected by a flow cytometer. **(D)** Cell viability was calculated as G2/M phase. Values are shown in percentage as compared to control cells. All data in bar charts represent mean ± SEM, *n* = 3. **p < 0.01, ***p < 0.001 represents G0/G1 phase vs. control group; ^##^*p* < 0.01 and ^###^*p* < 0.001 represents G2/M phase vs. control group.

To study the vulnerability of HT-22 cells to oxidative stress, HT-22 cells were exposed to various concentrations of H_2_O_2_ (200, 400, 600, and 800 μM) for 24 h, and the cell viability was detected following the H_2_O_2_ challenge using CCK-8 Kit as described previously (Zhang et al., 2018). Increase in H_2_O_2_ concentrations caused a dose-dependent decrease in cell viability as compared to control (Figures 1A,B). Observed results showed that 400 μM H_2_O_2_ significantly induced a decrease in cell density. To further investigate H_2_O_2_-induced oxidative injury, flow cytometer assay was performed to analyze cell-cycle distribution, and we found that cell cycle was arrested at the G2/M phase in the presence of H_2_O_2_ (Figures 1C,D), which is in accordance with a previous study (Liu H. et al., 2017). Moreover, exposure to increasing H_2_O_2_ concentrations resulted in a dose-dependent decrease in EdU incorporation to cells (Supplementary Figure 1). These results demonstrated that H_2_O_2_-mediated oxidative injury significantly inhibits cell proliferation.

### H_2_O_2_ Damages Mitochondria of HT-22 Cells

**Figure 2:**
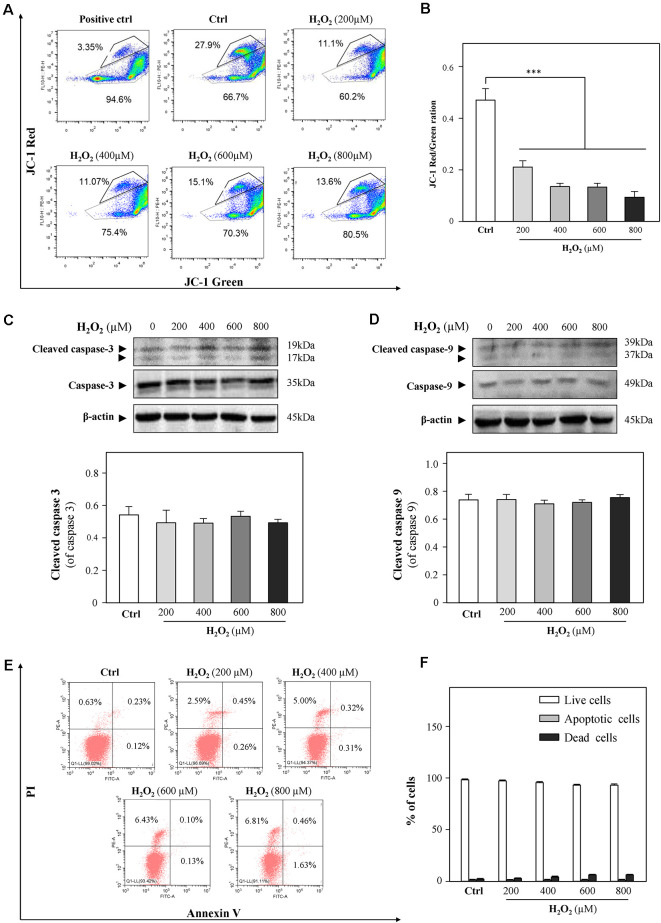
H_2_O_2_ shows impairment functions to mitochondria, but it cannot induce apoptosis in HT-22 cells. Cultured HT-22 cells were incubated with H_2_O_2_ at different concentrations (200–800 μM) for 24 h; control group (Ctrl) consists of the untreated cells. **(A)** Representative dot plot of the changed mitochondrial membrane potential using flow cytometry after labeling the fluorescent probe with JC-1. The changes in mitochondrial membrane potential induced by 10 μM CCCP were used as positive control. **(B)** Ratios of JC-1 red/green were shown in histograms. **(C)** Western blot analyses of cleaved-caspase 3 and caspase 3. Expression of β-actin was served as a loading control. Quantification plot of cleaved-caspase 3 was shown in histograms. **(D)** Western blot analyses of cleaved-caspase 9 at and caspase 9. Expression of β-actin served as a loading control. Quantification plot of cleaved-caspase 9 was shown in histograms. **(E)** Cell apoptosis was detected by flow cytometry with Annexin V/PI apoptosis detection kit using a flow cytometer. **(F)** Values are in percentage of apoptotic cell rates of live cells, apoptotic cells, and dead cells, as calibrated from **(E)**. Values are in percentage as compared to control (no drug treatment), and each point represents the mean ± SEM, *n* = 3. ***p < 0.001 vs. control group. CCCP, carbonyl cyanide m-chlorophenylhydrazone.

A previous study demonstrated that cardiac mitochondria are vulnerable to oxidative stress (Kim et al., 2016). In order to evaluate the mitochondrial functions of HT-22 cells in response to H_2_O_2_, mitochondrial membrane potential was detected by JC-1 staining. As shown in Figures 2A,B, H_2_O_2_ at different concentrations considerably decreased the red/green ratio, indicating that H_2_O_2_ caused mitochondrial dysfunctions. Since the change in mitochondrial membrane potential is an indicator for apoptosis, we next examined whether H_2_O_2_ could induce apoptosis of HT-22 cells. Western blot analysis showed that H_2_O_2_ did not trigger apoptosis, as indicated by the unchanged protein levels of cleaved caspase 3 and caspase 9 (Figures 2C,D). Moreover, there was no statistically significant difference of the percentages of apoptotic cells in the H_2_O_2_-treated group when apoptosis was analyzed by flow cytometer after Annexin V/PI staining (Figures 2E,F). These results suggested that the inhibitory effects of H_2_O_2_ observed in HT-22 cells were not through the induction of apoptosis.

### H_2_O_2_-Induced Oxidative Damage Is Due to Excessive Intracellular ROS Generation in HT-22 Cells

**Figure 3:**
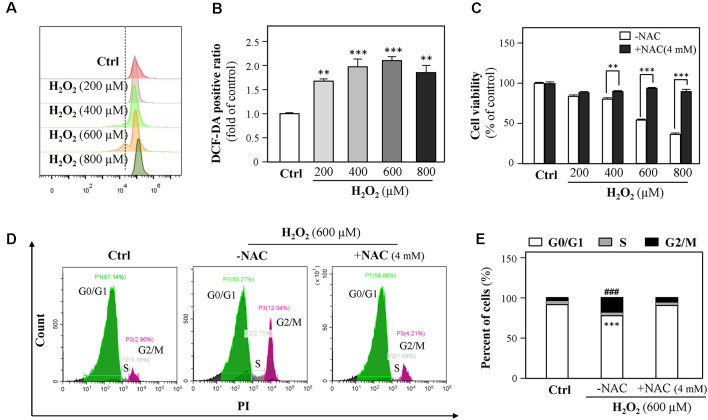
H_2_O_2_ increases intracellular reactive oxygen species (ROS) formation, thus causing cell-cycle arrest. **(A)** Cultured HT-22 cells were incubated with H_2_O_2_ at different concentrations (200–800 μM) for 24 h; the control group consists of the untreated cells. The amount of intracellular ROS was detected by DCFH-DA using a flow cytometry. **(B)** Mean fluorescence density of ROS level was calibrated from panel **(A)**. **(C)** Cultured HT-22 cells were treated with or without NAC (4 mM); simultaneously, the cells were treated with H_2_O_2_ at different concentrations (200–800 μM) for 24 h. The cell viability test was performed using CCK-8 kit, and the changes were shown in histograms as percentage with control. **(D)** Cultured HT-22 cells were seeded in six-well plates and incubated with H_2_O_2_ (600 μM) in the presence or absence with NAC (4 mM) for 24 h, respectively. The control group consists of the untreated cells. Cell apoptosis was detected by flow cytometry with Annexin V/PI apoptosis detection kit using a flow cytometer. **(E)** Values are in percentage of apoptotic cell rates of live cells, apoptotic cells, and dead cells, as calibrated from panel **(D)**. Values are shown in percentage; each point represents the mean ± SEM, *n* = 3. **p < 0.01, ***p < 0.001 and ^###^*p* < 0.001 vs. H_2_O_2_-treated group. NAC, *N*-acetylcysteine.

Because H_2_O_2_ is an ROS inducer, we next investigated the association between H_2_O_2_-mediated cell growth retardation and ROS generations. We found that exposure of HT-22 cells to H_2_O_2_ significantly elevated ROS levels (Figures 3A,B). Besides, cell loss induced by H_2_O_2_ (200, 400, 600, and 800 μM) was significantly prevented by the ROS scavenger, *N*-acetylcysteine (NAC, 4 mM, Figure 3C).

Since 600 μM H_2_O_2_ led to almost half HT-22 cell loss, this concentration was applied in the following experiments. HT-22 cells were treated with H_2_O_2_ in the presence or absence of NAC (4 mM) for 24 h and then the cell cycle was detected. Results showed that NAC apparently reduced the percentage of G2/M phase (Figures 3D,E). Together, these results suggested that H_2_O_2_-induced oxidative injury were mainly mediated by ROS generations.

### Nicotine Reduces H_2_O_2_-Induced Oxidative Damage

**Figure 4:**
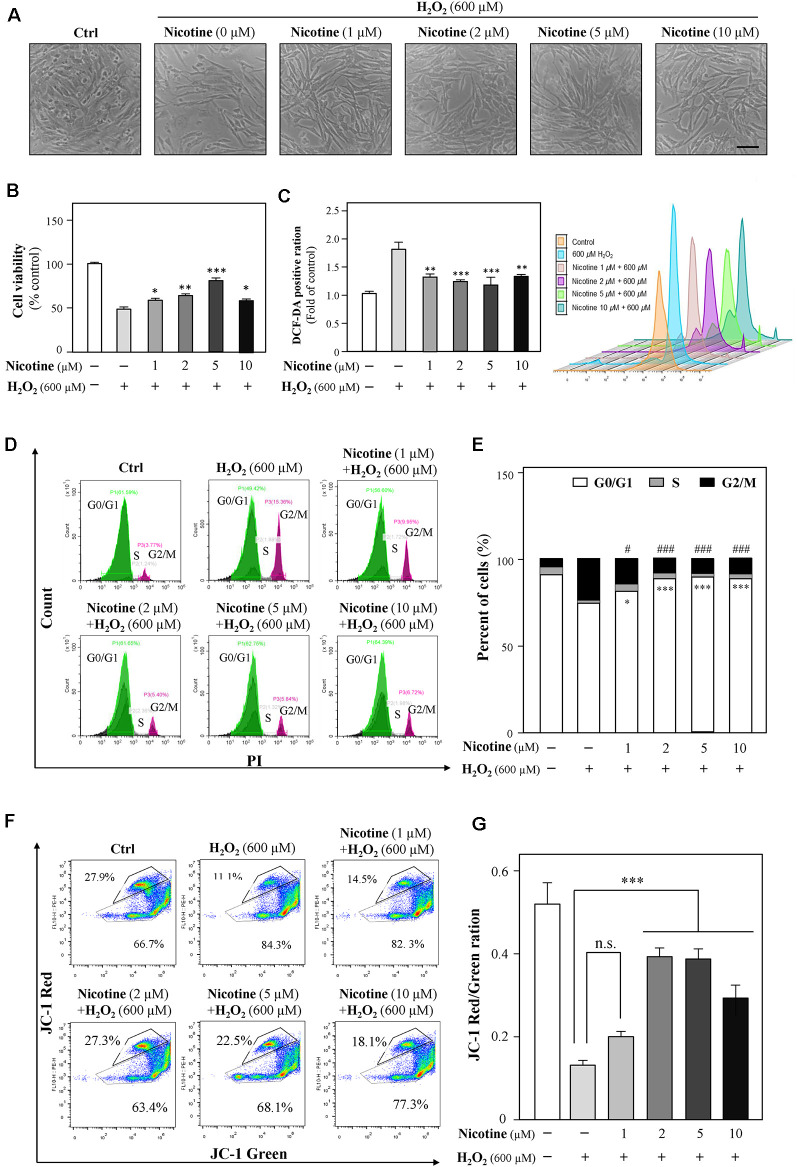
Nicotine suppresses the H_2_O_2_-induced oxidative injury in HT-22 cells. Cultured HT-22 cells were seeded in six-well plates and incubated in different concentrations of nicotine (1, 2, 5, and 10 μM) for 24 h, followed by the application of H_2_O_2_ (600 μM) for another 24 h; the control group consists of the untreated cells. **(A)** Representative images of the cell morphology. Bar = 100 μm. **(B)** Cell viability test was performed using CCK-8 kit, and the changes were shown in histograms as percentage with control. **(C)** The amount of intracellular ROS was detected by DCFH-DA using flow cytometry. Mean fluorescence density of the ROS level was calibrated. **(D)** The cell cycle was detected by flow cytometry with Annexin V/PI apoptosis detection kit using a flow cytometer. **(E)** Values are in percentage of apoptotic cell rates of live cells, apoptotic cells, and dead cells, as calibrated from panel **(D)**. **(F)** Representative dot plot of the changed mitochondrial membrane potential using flow cytometry after labeling the fluorescent probe with JC-1. **(G)** Ratios of JC-1 red/green were shown in histograms. All data in the bar charts represent mean ± SEM, *n* = 3. *p < 0.05, **p < 0.01, ***p < 0.001, and ^#^*p* < 0.05, ^###^*p* < 0.001 vs. control group. n.s., no significance.

Previously, a study showed neuroprotection of nicotine from Aβ-induced neurotoxicity (Rothbard et al., 2018). Here, we examined nicotine effects on HT-22 cell viability after the application of H_2_O_2_. HT-22 cells were pretreated with different concentrations (1, 2, 5, and 10 μM) of nicotine for 24 h. Afterward, the cells were exposed to 600 μM H_2_O_2_ for another 24 h. As shown in Figure 4 and Supplementary Figure 2, nicotine remarkably reduced H_2_O_2_-induced oxidative damage compared to the H_2_O_2_-treated group. Nicotine (1, 2, 5, and 10 μM) significantly increased the cell viability (Figures 4A,B), although the antioxidative functions of 10 μM nicotine showed a reduction. Besides, nicotine remarkably inhibited H_2_O_2_-stimulated ROS generation (Figure 4C). In addition, PI staining assay also showed that nicotine significantly recovered the cell cycle arrested by H_2_O_2_ (Figures 4D,E). Finally, the change in mitochondrial membrane potential was monitored and the results showed that the red/green ratio was significantly reduced by 600 μM of H_2_O_2_. However, pretreatment with nicotine substantially ameliorated the disruption of mitochondrial membrane potential induced by H_2_O_2_ (Figures 4F,G). Therefore, nicotine exerted its protective effects against H_2_O_2_ *via* reducing ROS production and restoring mitochondrial function and, as a result, facilitating cell proliferation.

### The Antioxidation of Nicotine Involves the Erk1/2 Signaling Pathway

**Figure 5:**
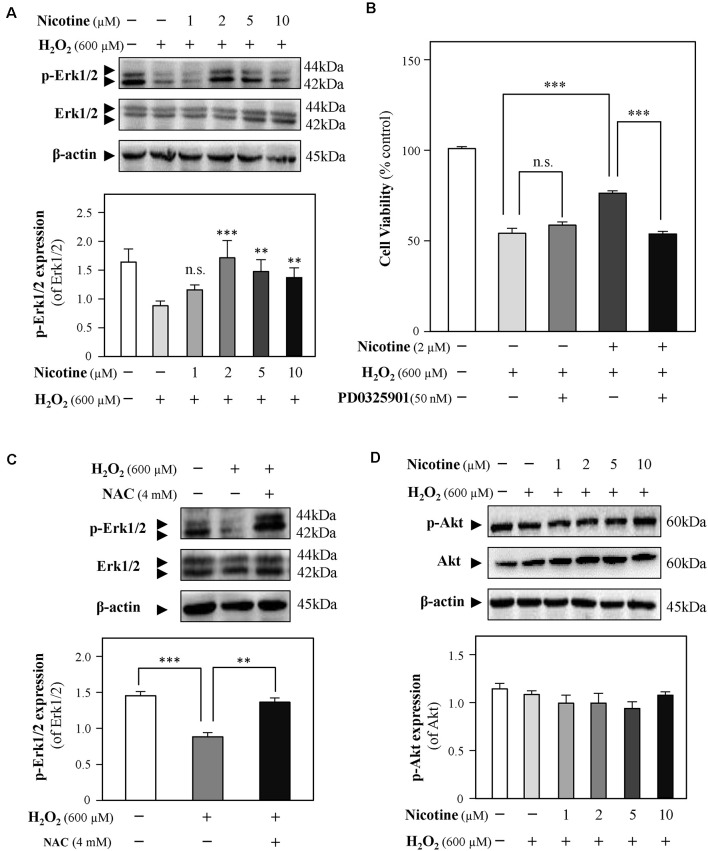
Nicotine reverses H_2_O_2_-induced oxidative damage through the Erk signaling pathway. Cultured HT-22 cells were treated with or without nicotine (1, 2, 5, and 10 μM) for 24 h, followed by H_2_O_2_ (600 μM) treatment in the presence or absence of PD0325901 (an inhibitor of Erk1/2, 50 nM) for 24 h or in the presence or absence of NAC (4 mM) for 24 h. The control group consists of the untreated cells. **(A)** The protein expressions of p-Erk1/2 and Erk1/2 were determined by western blotting (upper panel). Expression of β-actin served as loading control. The quantitation of p-Erk1/2 and Erk1/2 expressions was calibrated (lower panel). **(B)** Cell viability test was performed using CCK-8 kit, and the changes were shown in histograms as percentage with control. **(C)** The protein expressions of p-Erk1/2 and Erk1/2 were determined by western blotting (upper panel). Expression of β-actin served as loading control. The quantitation of p-Erk1/2 and Erk1/2 expressions was calibrated (lower panel). **(D)** The protein expressions of p-Akt and Akt were determined by western blotting (upper panel). Expression of β-actin served as loading control. The quantitation of p-Akt and Akt expressions was calibrated (lower panel). All data in bar charts represent mean ± SEM, *n* = 3. **p < 0.01, ***p < 0.001 vs. control group. NAC, *N*-acetylcysteine. n.s., no significance.

Both Erk1/2 and PI3K/Akt signaling pathways have been demonstrated to mediate neuroprotection from H_2_O_2_-mediated cell death (Fodero et al., 2004; Liu J. Y. et al., 2017; Wang T. et al., 2018). To investigate the molecular pathways underlying the effects of nicotine against H_2_O_2_-induced cell damage, the levels of phosphorylated Erk1/2 (p-Erk1/2) and Akt (p-Akt) upon H_2_O_2_ stimulation were monitored in the presence or absence of nicotine. Western blot analysis showed that phosphorylation of Erk1/2 was downregulated by H_2_O_2_ incubation in HT-22 cells; yet, nicotine treatment significantly increased phosphorylation of Erk1/2 (Figure 5A). To further explore the relation between H_2_O_2_ and Erk1/2, CCK8 assay was employed to assess HT-22 cell viability by treating with H_2_O_2_ and/or nicotine in the presence or absence of a selective Erk1/2 inhibitor (PD0325901). The results showed that the neuroprotection of 2-μM nicotine against H_2_O_2_ was totally abolished by PD0325901 (Figure 5B). Furthermore, we found that NAC also increased the level of p-Erk1/2 (Figure 5C), implying that ROS accumulation inactivates the Erk1/2 pathway. Taken together, these data suggested that the activated Erk1/2 pathway accounted for the neuroprotective functions of nicotine against H_2_O_2_-induced oxidative injury. In addition, our observation showed that p-Akt expression was not significantly influenced by H_2_O_2_ and nicotine (Figure 5D).

### α7 Nicotinic Acetylcholine Receptors Play a Dominant Role in the Antioxidation of Nicotine

**Figure 6:**
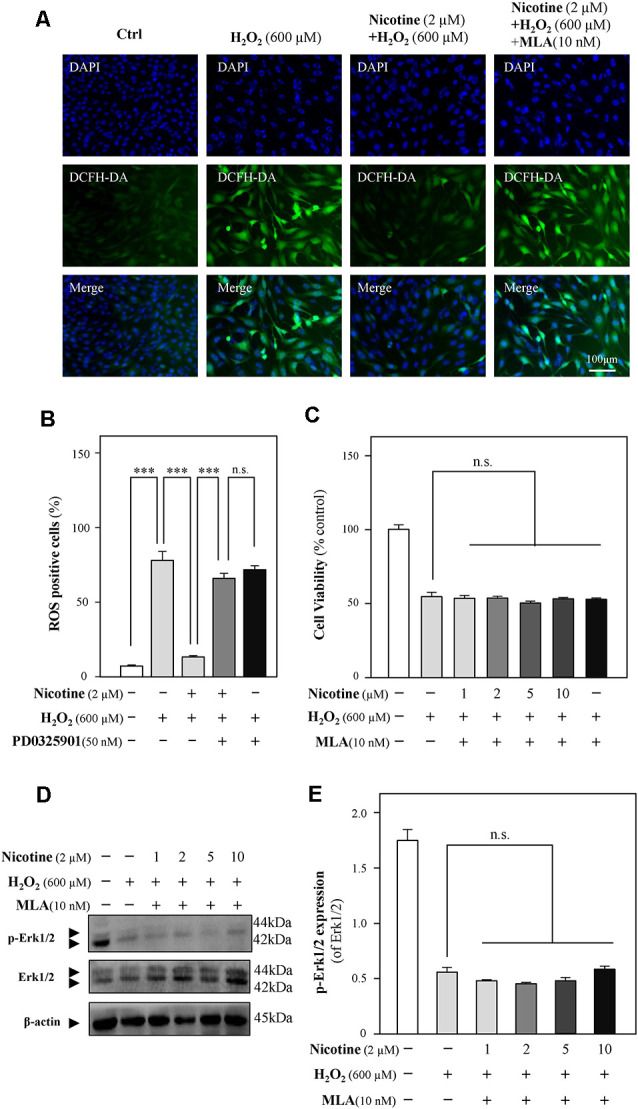
The Erk1/2 signaling pathway is activated by the binding of nicotine to α7-nAChRs. Cultured HT-22 cells were treated with or without nicotine (1, 2, 5, and 10 μM) for 24 h, followed by the application of H_2_O_2_ (600 μM) in the presence or absence of MLA (an inhibitor of α7-nAChRs, 10 nM) for another 24 h; the control group consists of the untreated cells. **(A)** Cells were stained with DCFH-DA. Cover glasses with cells were fixed and observed under a fluorescent microscope. Bar = 100 μm. **(B)** Mean fluorescence density of ROS level was calibrated. **(C)** Cell viability test was performed using CCK-8 kit, and the changes were shown in histograms as percentage with control. **(D)** The protein expressions of p-Erk1/2 and Erk1/2 were determined by western blotting (upper panel). Expression of β-actin served as loading control. **(E)** The quantitation of p-Erk1/2 and Erk1/2 expressions was calibrated from panel **(D)**. All data in bar charts represent mean ± SEM, *n* = 3. n.s., no significance. ***p < 0.001 vs. control group. MLA, methyllycaconitine citrate.

To further reveal the relationship between nicotine and Erk1/2, methyllycaconitine citrate (MLA), an α7-nAChR inhibitor, was applied to HT-22 cells together with H_2_O_2_ after the preincubation of nicotine. The neuroprotection of nicotine against ROS generation and cell-cycle arrest was almost prevented by MLA (Figures 6A,B, and Supplementary Figure 4). Cell viability analysis showed that the proliferation of nicotine against H_2_O_2_ was significantly inhibited by MLA (Figure 6C). Furthermore, we found that MLA fully inhibited p-Erk1/2 upregulation by nicotine (Figures 6D,E). Notably, the expression of α7-nAChRs was decreased by H_2_O_2_ (Supplementary Figure 3). These results suggest that α7-nAChRs is critical for nicotine-mediated activation of ERK1/2 signaling and antioxidation. In addition, we also analyzed the potential role of α4β2 nicotinic acetylcholine receptors (α4β2-nAChRs) in nicotine-mediated antioxidation. Our observation showed that α4β2-nAChRs did not influence the antioxidation of nicotine (Supplementary Figure 5).

## Discussion

Accumulating studies have shown that nicotine *in vitro* and *in vivo* promotes neuronal survival against oxidative stress (Guan et al., 2003; Liu and Zhao, 2004; Hritcu et al., 2017). Liu and Zhao (2004) previously reported that nicotine prevented Aβ-induced free radical in culture hippocampal neurons of rats. Our work provides a novel molecular link between nicotine and oxidative injury in HT-22 neuronal cells. The obtained results presented in the current study demonstrated that the pretreated nicotine at low concentrations (1, 2, 5, and 10 μM) inhibited H_2_O_2_-induced oxidative damage *via* activating its α7-nAChRs and subsequent Erk1/2 signaling pathway in HT-22 cells. These findings suggest that nicotine at low concentrations could be developed as a therapeutic agent for neurodegenerative disorders.

Oxidative stress has been implicated in numerous chronic or acute neurodegenerative disorders, such as AD (Behl et al., 1994) and PD (Fahn and Cohen, 1992), as well as neuroexcitotoxicity-related diseases (Coyle and Puttfarcken, 1993; Patel et al., 1996). The cytotoxic events mediated by oxidative stress are mainly due to the excessive ROS generations stimulated by H_2_O_2_ and the superoxide anion of free radicals. The accumulation of intracellular ROS has been linked to DNA damage, mitochondrial dysfunctions, lipid peroxidation, and protein destruction of neurons (Chen et al., 2012). H_2_O_2_ has been widely used as an *in vitro* inducer for oxidative stress to investigate neuroprotection in many different cell types (Desagher et al., 1997; Wang S. et al., 2018). In the present study, HT-22 cells were exposed to H_2_O_2_ for 24 h, and then intracellular ROS levels were remarkably increased, accompanied with a dose-dependent reduction of cell density. Furthermore, our results demonstrated that the decreased cell density mainly resulted from the changes of cell-cycle progression at the G2/M phase and the inhibition of cell proliferation caused by H_2_O_2_-stimulated ROS overproduction (Figure 1 and Supplementary Figure 1), which is consistent with previous studies that H_2_O_2_ inhibits cell proliferation in neural stem cells (Kim and Wong, 2009; Richter et al., 2015). Evidence has shown that 500 μM H_2_O_2_ significantly induced cell-cycle changes at the G2/M phase in neuroblastoma (B65) cells (Pizarro et al., 2009), which verified our results. On the other hand, Chen et al. (2000) reported that H_2_O_2_ induces human fibroblast cell apoptosis with an S-phase cell-cycle distribution. A possible explanation of the observation is that different cell types in response to oxidative stress could result in multiple characteristics of cell injury. Mitochondria are vulnerable to ROS (Sinha et al., 2013). Excessive ROS causes the loss of mitochondrial membrane potential and activates mitochondria-dependent neuronal cell deficits (Liu et al., 2015). We used the JC-1 probe to show that H_2_O_2_ induced a significant reduction in mitochondrial membrane potential (Figure 2A). Since mitochondrial depolarization has been suggested to be a requirement for cell apoptosis (Ankarcrona et al., 1995; Heiskanen et al., 1999), we further explored whether oxidative injury is related to cell apoptosis. Moreover, we found that apoptosis was not involved in H_2_O_2_-induced HT-22 cell oxidative injury (Figures 2C–F), which is in accordance with the study demonstrated by Xu et al. (2014). On the contrary, evidence clearly demonstrates that oxidative stress induces HT-22 cell injury *via* apoptosis or ferroptosis (Yoo et al., 2017; Yeo et al., 2019). Characteristics of cell injury in response to oxidative stress in HT-22 cells shown in these studies suggest oxidative stress-induced neuronal injury is related to multiple molecular mechanisms. In the current study, we confirmed that H_2_O_2_-induced oxidative damage mainly changes the cell cycle, inhibits cell proliferation, and triggers mitochondrial depolarization.

Many studies have described the neuroprotective effects of nicotine, including promoting newborn neuron survival in adult olfactory bulb (Mechawar et al., 2004), ameliorating dopamine neuron damage (Liu et al., 2012), decreasing NMDA-mediated neuroexcitotoxicity (Gahring et al., 2003), and preventing Aβ-mediated neurotoxicity (Fodero et al., 2004; Yu et al., 2011). In this study, we explored the potential protective ability of nicotine on H_2_O_2_-induced oxidative damage in HT-22 cells. We observed that pretreated nicotine at low concentrations (1, 2, 5, and 10 μM) could significantly decrease oxidative damages including increasing cell viability, recovering the cell cycle from G2/M phase arrest, and preventing mitochondrial dysfunctions *via* inhibiting ROS generations (Figure 4). However, the antioxidative abilities of 10-μM nicotine against H_2_O_2_ showed a decrease. The result further indicated that overdose of nicotine might result in side effects on CNS, such as nicotine addiction. Overdose of nicotine has shown a significant decrease in the neuronal densities and the increase in excitotoxicity in the hippocampus (Ferrea and Winterer, 2009). Our results highlight the robust neuroprotection of the application of nicotine at low concentrations.

The underlying mechanisms of nicotine against oxidative damages including alternation of cell cycle, inhibition of cell proliferation, and mitochondrial dysfunctions in HT-22 cells remain to be identified. Therefore, the extracellular signaling pathway by the application of nicotine was investigated. Nicotine exerts its function mainly *via* activating nicotinic acetylcholine receptors (nAChRs; Dani and Heinemann, 1996). Among different nAChRs, the α4/β2 receptors and α7 receptors have been shown to play a dominant role in the CNS (Hsu et al., 1997; Fodero et al., 2004; Liu et al., 2009; Lewis et al., 2018; Xu et al., 2019). Hence, we considered whether the neuroprotective effects of nicotine against oxidative damage could be mediated by the activation of α4/β2-nAChRs or α7-nAChRs, and then antioxidative effects of α4/β2-nAChRs which respond to nicotine in HT-22 cells were determined. As shown in Supplementary Figure 4, dihydro-β-erythroidine hydrobromide (DHβE), an antagonist of α4/β2 receptor, hardly influenced the neuroprotective effects of nicotine. However, we found that the antioxidative effects of nicotine could be abolished by the α7-nAChR-selective antagonist MLA, implying that α7-nAChRs play a critical role in HT-22 cell oxidative injury (Figure 6). Literatures have documented that nicotine acts its neuroprotective functions on oxidative stress *via* the activation of α7-nAChRs *in vitro* and *in vivo*, for example in astrocytes of mice, hippocampal cells of rats, and PC12 cells (Guan et al., 2003; Liu and Zhao, 2004; Liu et al., 2015; Hritcu et al., 2017). We first evaluated the potential role of α7-nAChRs in the regulation of oxidative stress in HT-22 cells. An agonist of α7-nAChRs, GTS-21, significantly prevented neuroinflammation in mice (Nullens et al., 2016), which further suggests that the activation of α7-nAChRs plays a key role in neuroprotection. Moreover, the activation of α7-nAChRs promotes neuronal survival against Aβ-induced neurotoxicity *via* suppressing apoptosis in SH-SY5Y cells (Xu et al., 2019). Taken together, these investigations implied that an agonist of α7-nAChRs could be developed as a therapeutic agent against neurodegeneration.

Multiple mechanisms were involved in the neuroprotective effects of nicotine, such as inhibition of astrocyte activation, PI3K signaling pathway, apoptotic signaling pathway, Wnt/β-catenin signaling pathway, and Erk1/2 signaling pathway (Fodero et al., 2004; Yu et al., 2011; Liu et al., 2012; Lombardo and Maskos, 2015). The phosphorylation of Erk1/2 (p-Erk1/2) is known to be induced by nicotine *in vivo* (Brunzell et al., 2003) and *in vitro* (Dajas-Bailador et al., 2002), implying that nicotine might activate α7-nAChRs to initiate the signaling cascade that results in Erk phosphorylation. In the current study, nicotine significantly upregulated p-Erk1/2 in HT-22 cells (Figure 5). Upregulated p-Erk1/2 was significantly abolished by the application of an α7-nAChR antagonist, MLA (Figures 6D,E). Besides, an Erk1/2 inhibitor, PD0325901, remarkably abolished the neuroprotective effects of nicotine. These findings suggest that the activation of the α7-nAChRs/Erk1/2 signaling pathway contributes to the neuroprotection of nicotine on oxidative injury in HT-22 cells. Previous study showed that the Erk1/2 signaling pathway could be activated by nicotine *via* α7-nAChR activation in SH-SY5Y cells and rat hippocampal cells (Dajas-Bailador et al., 2002), which is consistent with our work. Similarly, α7-nAChR activation protected from 1-methyl-4-phenylpyridinium-induced cell apoptosis *via* the Erk/p53 signaling pathway in SY5Y cells (Xu et al., 2019). Evidence, on the contrary, demonstrated that exposure of α7-nAChRs to nanomolar Aβ42 stimulated Erk2 MAPK cascade in the hippocampus of Tg2576 mice carrying a human APP transgene with K670N-M671L mutation (Dineley et al., 2001), indicating that the α7-nAChRs/Erk2 signaling pathway might mediate neurodegeneration. However, the specific nAChR involved and its potential role in neuroprotective and neurodegenerative effects remain to be clarified.

Additionally, nicotine activated Erk1/2 phosphorylation *via* CaMKII or glutamate receptor rather than binding to its α7-nAChRs in cultured primary cortical neurons (Steiner et al., 2007; Chen et al., 2018). One possible reason leading to such inconsistency might be the profound different distribution of nAChRs in the hippocampus and cortex (Séguéla et al., 1993; Picciotto et al., 1995). Furthermore, it has been reported that α3/β4-nAChR is responsible for Erk1/2 phosphorylation in PC12 cells (Nakayama et al., 2006). However, Rebecca and colleagues showed that α-conotoxin Au1B, a specific antagonist of α3/β4-nAChRs, could not inhibit Erk1/2 phosphorylation (Steiner et al., 2007), indicating that α3/β4-nAChRs might be insufficient to activate Erk1/2. Here, we first found that nicotine could have neuroprotective effects in HT-22 cells from oxidative injury through α7-nAChR activation and then promote the Erk1/2 signaling pathway. In addition, the PI3K/Akt signaling pathway has also been implicated in nicotine-mediated neuroprotection (Steiner et al., 2007; Takeuchi et al., 2009; Huang et al., 2012). However, we found that the PI3K/Akt signaling pathway was not influenced by the application of either H_2_O_2_ or nicotine (Figure 5D). Besides, LY294002, a PI3K/Akt inhibitor, could not inhibit neuroprotection of nicotine (data not shown). Consistent to our data, the PI3K/Akt signaling pathway was not involved in nicotine’s neuroprotective activation against 1-methyl-4-phenylpyridinium-induced cell apoptosis in SH-SY5Y cells (Xu et al., 2019).

Nicotine can be physiologically metabolized to nornicotine in CNS in human. Specifically, nornicotine was shown to reduce soluble Aβ peptide aggregation based on alteration of amyloid folding (Dajas-Bailador et al., 2000). This suggests a purported ability of nicotine as a neuroprotective agent. So, the potential efforts of nicotine in the application of oxidative-induced neuronal injury could be further explored.

In conclusion, outcomes show that nicotine exerts its neuroprotection against H_2_O_2_-induced oxidative injury *via* activating the α7-nAChRs/Erk1/2 signaling pathway in HT-22 cells, which could provide new mechanistic insights into the role of nicotine in oxidative stress. The low dose of nicotine could be developed as a novel therapeutic strategy in oxidative stress-related neurodegenerative disorders, such as AD and PD.

## Data Availability Statement

All datasets presented in this study are included in the article/Supplementary Material.

## Author Contributions

YD and WB performed most of the experiments. YD conceived the project and designed the experiments. KZ, SW, YX, and BL participated in data analysis. All authors contributed to the article and approved the submitted version.

## Conflict of Interest

The authors declare that the research was conducted in the absence of any commercial or financial relationships that could be construed as a potential conflict of interest.
