# Supplementary material for: Nicotine Prevents Oxidative Stress-Induced Hippocampal Neuronal Injury Through α7-nAChR/Erk1/2 Signaling Pathway
Source: Front Mol Neurosci. 2020 Nov 12;13:557647. doi: 10.3389/fnmol.2020.557647 (PMC7717967; doi:10.3389/fnmol.2020.557647)
Supplement: Supplementary file 3 [file Data_Sheet_1.docx]

Supplementary Data


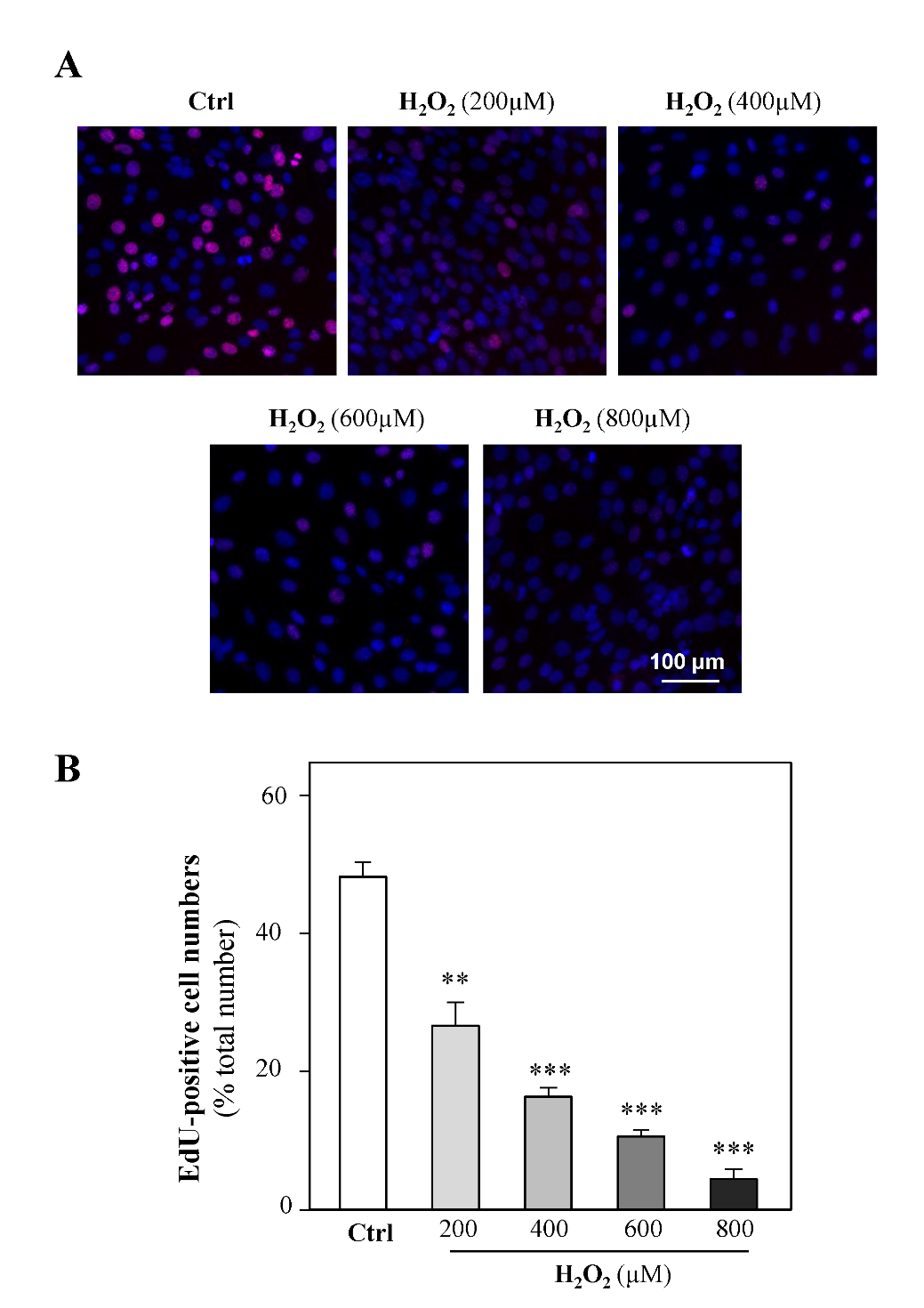


**Figure S1.** The different concentrations of H_2_O_2_ inhibits cell proliferation. (A) Representative images. (B) Quantitative measurement of EdU incorporation to cells after all treatments. Scale bar, 100 µm. All data in the bar chart represent mean ± SEM of 3 independent experiments. ^**^ *p* < 0.01 and ^***^ *p* < 0.001 versus control group.

**
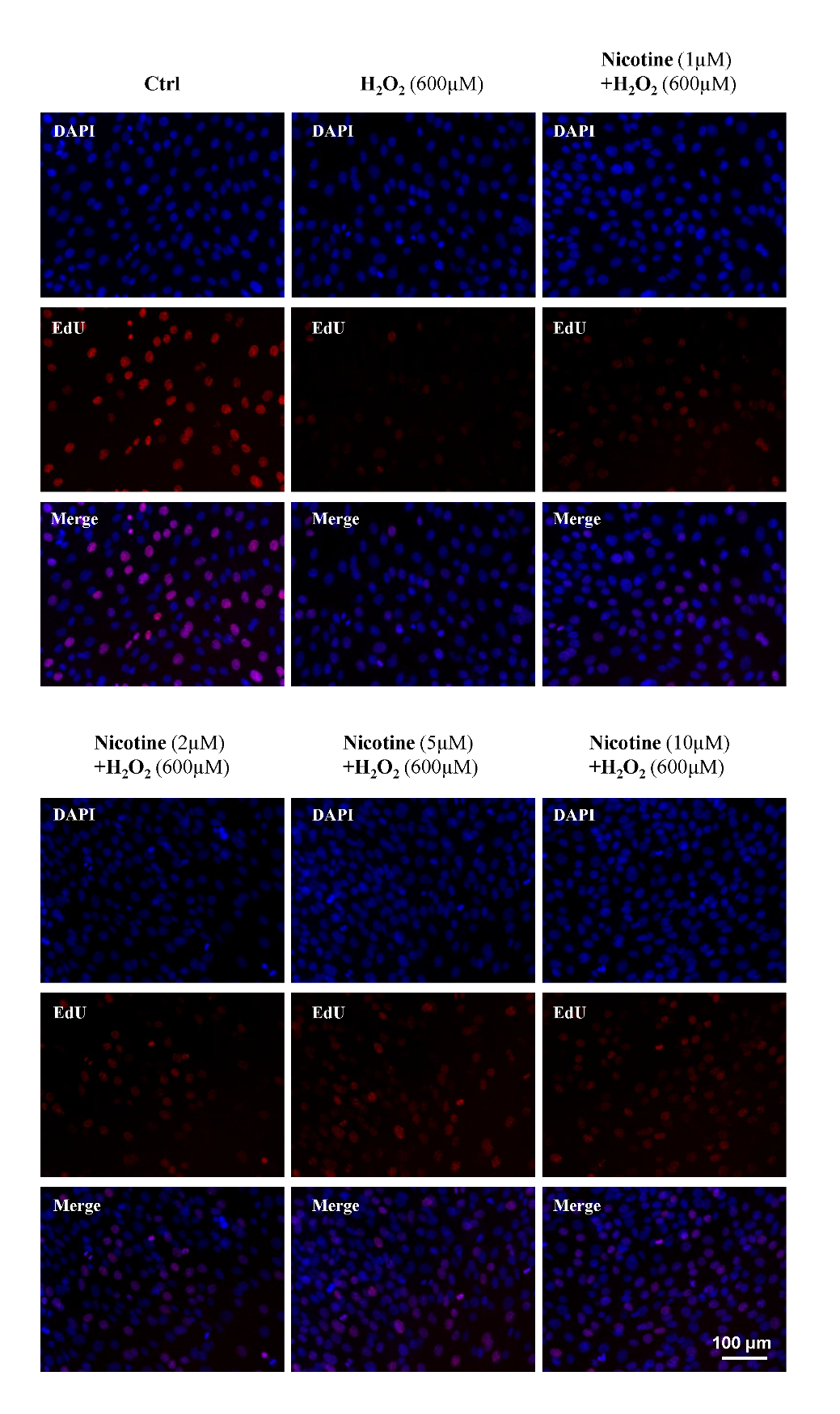
**

**Figure S2.** Nicotine at different concentrations (1, 2, 5, or 10 *μ*M) recovers cell proliferation from H_2_O_2_-mediated oxidative injury. Representative images of each treated group. The first and fourth lines showed cells by DAPI staining. The second and fifth lines showed cells by EdU staining. Scale bar, 100 µm.


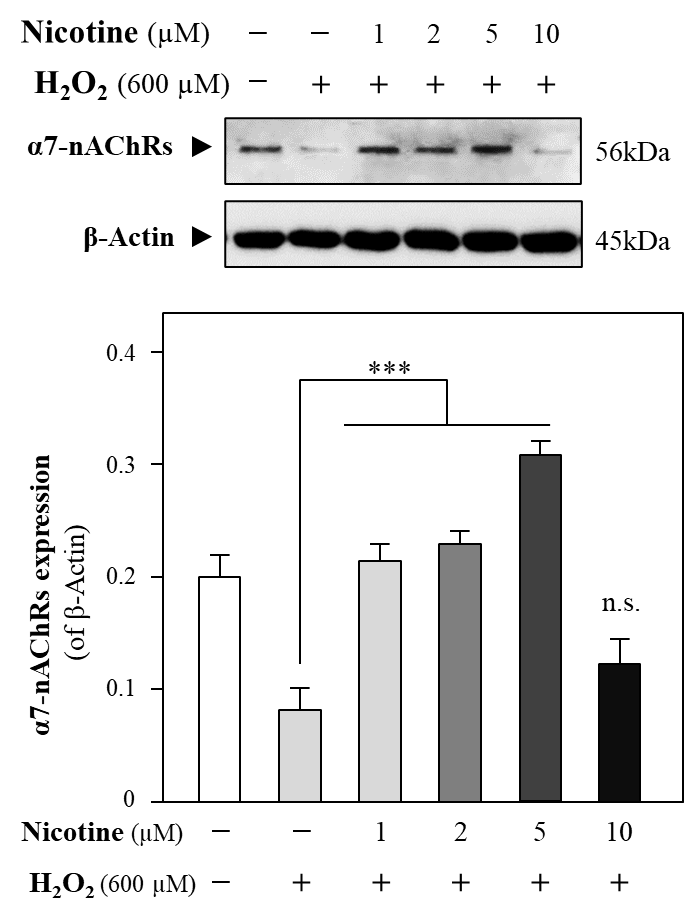


**Figure S3.** Cultured HT-22 cells were treated with or without nicotine (1, 2, 5, 10 µM) for 24 h, following by the application of H_2_O_2_ (600 µM) for another 24 h; control group was the un-treated cells. The protein expressions of α7-nAChRs were determined by western blotting (upper panel). Expression of β-actin served as loading control. The quantitation of α7-nAChRs expressions were calibrated. All data in bar charts represent mean ± SEM, n = 3. n.s., no significance; ** *p* < 0.01, *** *p* < 0.001 versus H_2_O_2_-treated group.


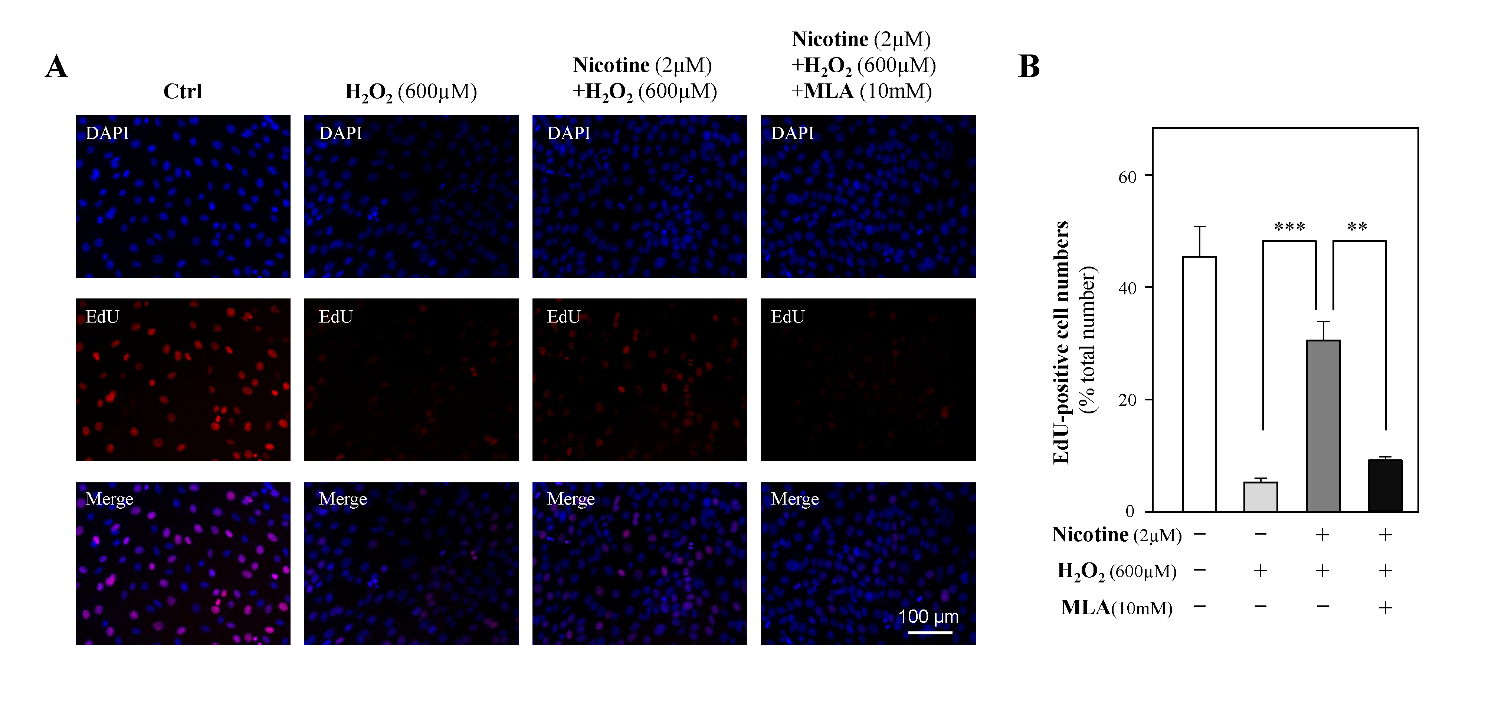


**Figure S4.** Nicotine recovered cell proliferation is completely prevented by MLA, an inhibitor of α7-nAChRs. (A) Representative images. (B) Quantitative measurement of EdU incorporation to cells after all treatments. Scale bar, 100 *µ*m. All data in the bar chart represent mean ± SEM of 3 independent experiments. ^**^ *p* < 0.01, ^***^ *p* < 0.001 versus nicotine-neuroprotective group.


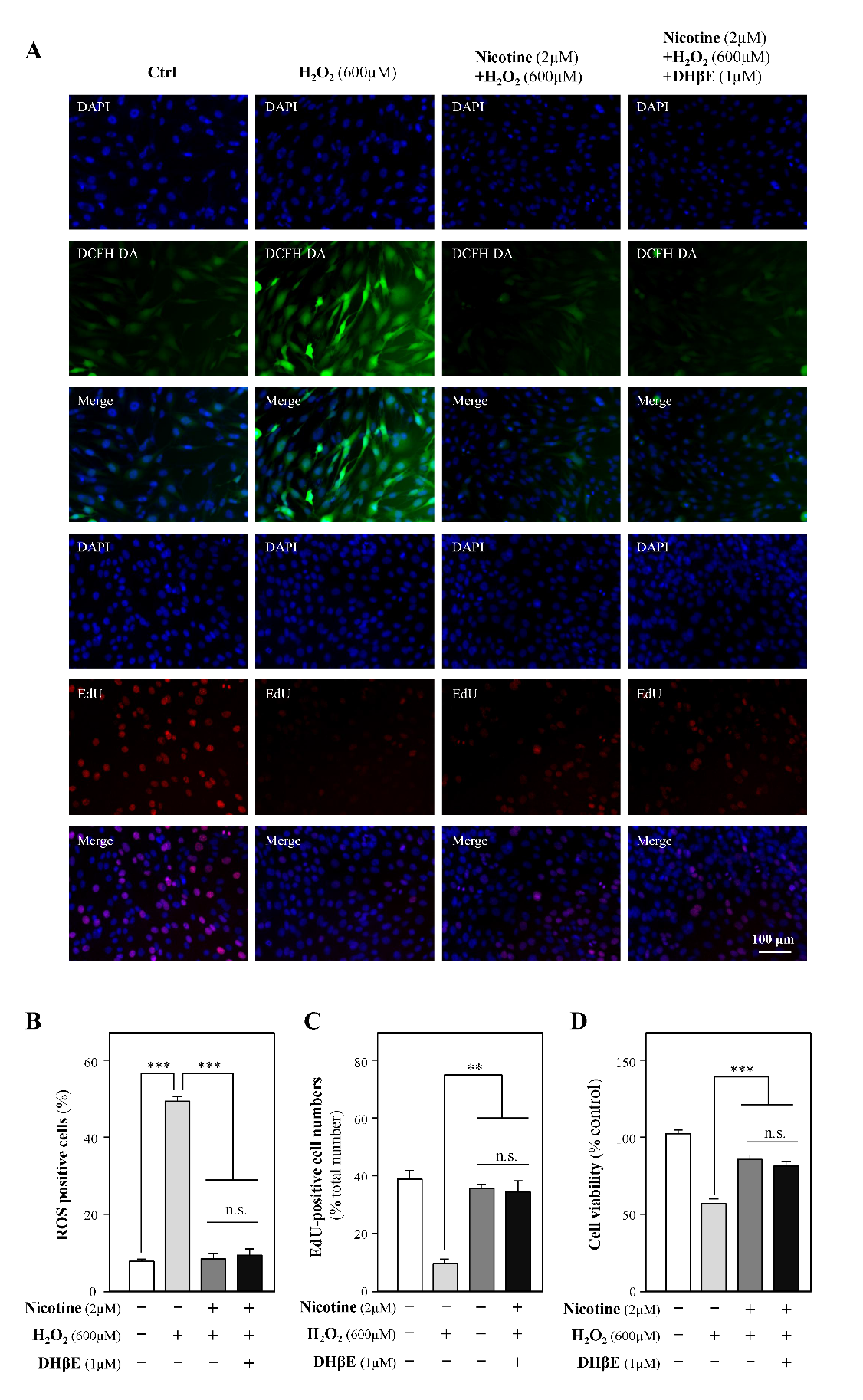


**Figure S5.** Α4β2-nAChRs are not essential for the neuroprotection of nicotine against H_2_O_2_-induced oxidative stress. Representative images showed (**A**) ROS-positive cells and cell proliferation after each treatment. (**B**) Quantification of ROS-positive cells. (**C**) Quantitative measurement of EdU incorporation to cells. (**D**) Cell viability. Scale bar, 100 µm. All data in bar charts represent mean ± SEM of 3 independent experiments. Control group was vehicle-treated cells. n.s., no significance; ^**^ *p* < 0.01 and ^***^ *p* < 0.001 versus H_2_O_2_-treated group. DHβE, Dihydro-β-erythroidine hydrobromide.
